# Supplementary material for: A Global Screen for Assembly State Changes of the Mitotic Proteome by SEC-SWATH-MS
Source: Cell Syst. 2020 Feb 26;10(2):133–155.e6. doi: 10.1016/j.cels.2020.01.001 (PMC7042714; doi:10.1016/j.cels.2020.01.001)

Q13523 | PRP4B_HUMAN | PRPF4B KIAA0536 PRP4 PRP4H PRP4K
Monomer MW [kDa]: 116.987 Monomer expected elution fraction: 39

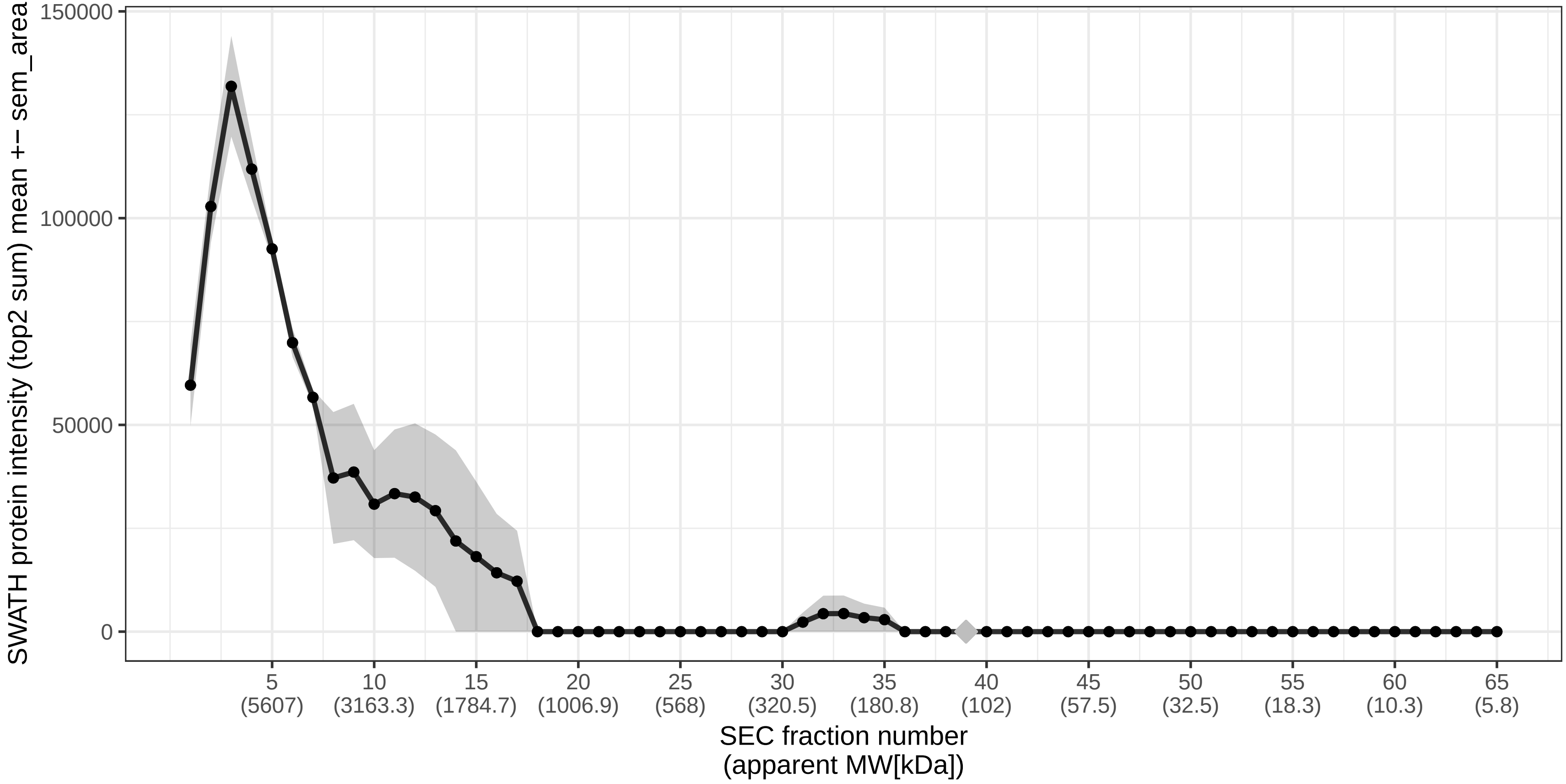

Supplement: Data S1. SEC-SWATH-MS Protein Chromatograms, Related to Figure 1 [file mmc6.zip › SECchrom_Q13523_PRP4B_HUMAN_PRPF4B_KIAA0536_PRP4_PRP4H_PRP4.pdf]
